# Clinical application of regional and intermittent hepatic inflow occlusion in laparoscopic hepatectomy

**DOI:** 10.3389/fonc.2022.1026274

**Published:** 2022-10-06

**Authors:** Longqing Shi, Baoyang Luo, Yong Yang, Yurong Miao, Xin Li, Donglin Sun, Qiang Zhu

**Affiliations:** ^1^ Department of Hepatobiliary and Pancreatic Surgery, Third Affiliated Hospital of Soochow University, Changzhou, China; ^2^ Department of Hepatobiliary Surgery, Taizhou People’s Hospital, Taizhou, China; ^3^ Department of General Surgery, Children’s Hospital of Nanjing Medical University, Nanjing, China

**Keywords:** laparoscopic hepatectomy, liver surgery, Pringle’s maneuver occlusion, regional occlusion, intraoperative bleeding

## Abstract

**Aim:**

The aim of this study is to investigate the advantages and disadvantages of regional and intermittent hepatic inflow occlusion in laparoscopic hepatectomy.

**Methods:**

The clinical data of 180 patients who underwent laparoscopic liver surgery in Taizhou People’s Hospital from 2015 to 2021 were analyzed retrospectively. The patients were divided into the regional occlusion group (*n* = 74) and the Pringle’s maneuver occlusion group (*n* = 106) according to the technique used in the intraoperative hepatic inflow occlusion. The pre- and intra-operative indicators, postoperative recovery indicators, and complications of the two groups were compared.

**Results:**

There were no significant differences (*p* > 0.05) between the groups in terms of sex, age, preoperative alanine aminotransferase (ALT), preoperative aspartate aminotransferase (AST), preoperative albumin, alpha-fetoprotein, liver cirrhosis, hepatitis B, tumor location, gas embolism, intraoperative blood transfusion, postoperative albumin, postoperative total bilirubin (TBIL), postoperative hospital stays, and complications. The preoperative TBIL and operation time were higher in the regional occlusion group than in the Pringle’s maneuver occlusion group, while the amount of intraoperative bleeding, postoperative ALT, and AST in the regional occlusion group were significantly lower than those in the Pringle’s maneuver occlusion group (*p* < 0.05).

**Conclusion:**

The two occlusion techniques are equally safe and effective, but regional hepatic inflow occlusion is more advantageous in operation continuity, intraoperative bleeding, and postoperative liver function recovery. The long duration and high precision of the regional blood flow occlusion technique demands a more experienced physician with a higher level of operation; therefore, it can be performed by experienced laparoscopic liver surgeons.

## Introduction

Since the first laparoscopic liver resection was performed in 1991, the application of laparoscopic techniques in liver surgery has gradually unfolded, and laparoscopic liver resection has the advantages of less bleeding, less trauma, and faster recovery for almost all types of liver resection (1), ranging from partial hepatectomy to liver transplantation (2). Currently, the scope of surgery has evolved from the initial partial hepatectomy to the current accurate lobe hepatectomy and segmental hepatectomy (3).

The most dangerous complications of laparoscopic hepatectomy are massive hemorrhage and CO_2_ gas embolism (4). Massive hemorrhage during a surgical operation will increase postoperative morbidity and mortality and is a major reason for conversion from laparoscopy to open surgery (5). Hepatic inflow occlusion is an effective method to control bleeding during hepatectomy. The most commonly used technique is the total hepatic inflow occlusion (Pringle’s maneuver) (6). Pringle’s maneuver was first described in 1908. It has the characteristics of wide application, simple operation, and favorable occlusion effect, but its disadvantages have also been noticeable. The occlusion of all hepatic blood flow into the first hepatic hilum with intermittent clamping and release is required for Pringle’s maneuver. Thus, the continuity of the operation is interrupted, and bleeding of the hepatic cutting surface is increased significantly during the intermittent period. With the development of surgical technology, the application of regional occlusion has increased. Regional occlusion only blocks blood supply in the liver segment, does not affect the blood supply in the remnant liver, ensures the continuity of the operation, and reduces the amount of intraoperative bleeding. However, a demanding surgical technique is required for regional occlusion. At present, the regional occlusion approaches mainly include the intraglissonian and extraglissonian approaches. Both approaches can block the corresponding blood flow of the hepatic lobe or segment to achieve the purpose of accurate segmental hepatectomy. Some of the liver centers not only occlude hepatic blood flow into the first hepatic hilum, but also further dissect and block the hepatic vein at the second hepatic hilum to further reduce the incidence of surgical bleeding and CO_2_ embolism.

The first hepatic hilum contains many blood vessels and bile ducts, where the space is narrow, and the anatomical structure is complex. If the surgeon is not careful enough to deal with the small branches of the portal vein during the surgical operation, it can result in uncontrollable bleeding and increase the incidence of complications. Thus, we explored the feasibility of the regional inflow occlusion technique by retrospectively comparing and analyzing the surgical results of the Pringle’s maneuver and the regional inflow occlusion technique.

## Materials and methods

General data: The clinical data of patients who underwent laparoscopic liver surgery of all types in Taizhou People’s Hospital from 2015 to 2021 were retrospectively analyzed. The patients included 101 men and 79 women with a mean age of 56.04 ± 12.44 years, all with preoperative liver function scores of Child–Pugh A−B and the final diagnosis was confirmed by postoperative pathology.

Patient inclusion criteria: (1) preoperative imaging confirmed as a liver disease; (2) preoperative liver function score was Child–Pugh class A or B; (3) postoperative pathological diagnosis of liver disease; (4) no combined surgery involving other organs; and (5) no history of open upper abdominal surgery. Patient exclusion criteria: (1) tumor invading the hilar area of the liver; (2) comorbid severe cardiopulmonary disease; (3) comorbid hematological disease; and (4) conversion from laparoscopy to laparotomy.

Surgical procedure: (1) When the pneumoperitoneum pressure was stabilized to 12 mmHg, a 12-mm trocar was inserted into the abdominal cavity through a subumbilical longitudinal incision. A 5-mm trocar was placed at the intersection of the left midclavicular line and the transverse umbilical line, and another 5-mm trocar was positioned at the intersection of the left midclavicular line and subcostal margin line for assistance. A 5-mm trocar immediately subcostal in the right midclavicular line and a 12-mm trocar in the right midclavicular line were established as the main operation ports. The exact position was adjusted according to the patient’s size, the size of the liver, and the location of the liver resection. (2) The perihepatic ligament was severed, and the ligamentum teres hepatitis, falciform ligament, left coronary ligament, and left triangular ligament were cut off in turn; thereafter, the right coronary ligament and hepatorenal ligament were severed. (3) Hepatic inflow occlusion: (a) Regional hepatic inflow occlusion: First, the proper hepatic artery and portal vein were dissected, and the arteries and veins of each hepatic lobe were dissected along the proper hepatic artery and portal vein. It is worth noting that some patients have a middle hepatic artery, which should be carefully identified during the surgical operation to avoid poor occlusion effects. In addition, before dissecting hepatic artery branches and portal vein branches, it is necessary to confirm the existence of other hepatic artery and portal vein branches to avoid injuries due to vascular variations in some patients. (b) Pringle’s maneuver: The hepatoduodenal ligament was dissected, exposed, and routinely clamped using a tape for 15 min every period, and then released for a 5-min interval.

Diagnostic criteria for CO_2_ gas embolism: Intraoperative monitoring of patient vital signs included the arterial oxygen saturation (SPO_2_) measured by finger pulse oximetry and the partial pressure of end-tidal CO_2_ (PETCO_2_). CO_2_ gas embolism was diagnosed if the patient had a rapid drop in PETCO_2_, a drop in blood pressure when severe, a rapid increase in heart rate, or even a drop in oxygen saturation.

Statistical analysis: SPSS 19.0 software was used for statistical analysis. Measurement data with a normal distribution were presented as mean ± standard deviation (SD). Statistical differences were determined using analyses of variance or Student’s *t*-tests. The qualitative data were analyzed using the Chi-square and Fisher’s exact test. Differences were considered statistically significant when *p* < 0.05.

## Results

### The comparison of disease composition between two groups of patients

To study the relationship between clinical indicators and surgery-related outcomes of patients,180 patients with various types of liver diseases were included in this study, including 91 cases of hepatocellular carcinoma (HCC), 8 cases of hepatolithiasis, 37 cases of hepatic hemangioma (HH), 11 cases of focal nodular hyperplasia (FNH), 6 cases of metastatic hepatic carcinoma, 13 cases of intrahepatic cholangiocarcinoma (ICC), 4 cases of hepatic adenoma, 3 cases of inflammatory pseudotumor, 2 cases of coagulative necrosis, 2 cases of inflammatory myofibroblastic tumor of the liver (IMT), and 3 cases of intrahepatic choledochal cyst. A detailed information is presented in **Table 1**. There was no significant difference in disease composition between the two groups (*p* > 0.05).

**Table 1:** The comparison of the disease composition of patients between groups with different inflow occlusion techniques.

LLR				
	Regional occlusion(*n* = 74)	Pringle’s maneuver occlusion(*n* = 106)	*χ* ^2^	*p*-value
Diagnosis			9.709	0.452
HCC	35	56		
Hepatolithiasis	6	2		
HH	13	24		
FNH	6	5		
ICC	5	8		
Hepatic adenoma	3	1		
Metastatic hepatic carcinoma	2	4		
Inflammatory pseudotumor	2	1		
IMT	1	1		
Coagulative necrosis	0	2		
choledochal cyst	1	2		

### The comparison of preoperative conditions between the two groups

A total of 180 patients were retrospectively analyzed, including 101 men and 79 women, with an average age of 56.04 ± 12.44 years. The patients were divided into two groups: the regional hepatic inflow occlusion group (*n* = 74) and Pringle’s maneuver occlusion group (*n* = 106). A statistical analysis of sex, age, alanine aminotransferase (ALT), aspartate aminotransferase (AST), total bilirubin (TBIL), alpha-fetoprotein (AFP), hepatitis B surface antigen, and liver cirrhosis between the two groups was performed. There were no significant statistical differences in the general information of the patients except in preoperative TBIL between the two groups, as presented in **Table 2**.

**Table 2:** The comparison of preoperative conditions between the two groups of patients with different inflow occlusion techniques.

LLR				
	Regional occlusion(*n* = 74)	Pringle’s maneuver occlusion(*n* = 106)	*t*/*χ* ^2^	*p*-value
Age (years)	54.99 ± 13.66	56.78 ± 11.52	0.953	0.342
Sex			0.025	0.873
Men	41	60		
Women	33	46		
ALT (U/L)	45.85 ± 86.74	28.56 ± 22.34	1.676	0.098
AST (U/L)	39.48 ± 52.66	28.37 ± 13.39	1.777	0.079
AFP (ug/L)	100.98 ± 311.64	67.28 ± 236.03	0.825	0.410
Cirrhosis			0.737	0.391
Positive	24	41		
Negative	50	65		
TBIL (μmol/L)	18.03 ± 9.61	14.34 ± 5.71	2.960	0.004
ALB (g/L)	40.28 ± 6.70	41.40 ± 4.65	1.332	0.185
HbsAg			0.450	0.502
Negative	49	65		
Positive	25	41		

### The comparison of intraoperative conditions between the two groups

To analyze and compare the surgical outcomes of the two surgical approaches, statistical analysis was performed on the perioperative general characteristics of patients, including tumor location, operative time, hemorrhage, blood transfusions, and gas embolism in this study. The results revealed that the operative time in the regional occlusion group was longer than that in the Pringle’s maneuver occlusion group (239.23 ± 96.26 vs. 204.93 ± 89.62 min), the difference was statistically significant (*t* = 2.450, *p* = 0.015), and the amount of intraoperative bleeding was less in the regional occlusion group than in the Pringle’s maneuver occlusion group (209.05 ± 181.28 vs. 292.92 ± 309.69 ml, *t* = 2.284, *p* = 0.024). There was no occurrence of gas embolism in the regional hepatic inflow occlusion group, while there were two cases in the Pringle’s maneuver occlusion group with no statistically significant difference between the two groups (*χ* ^2^ = 0.217, *p* = 0.641). One case of CO_2_ gas embolism was caused by intraoperative injury to the short hepatic vein. In the other case, gas embolism was derived from the “aspiration” of air bubbles into the right atrium through the fissure of the injured hepatic vein into the inferior vena cava after the opening of the sieve-plate fenestrae in the middle hepatic vein of the hepatic cutting surface due to asynchronous blockage of the hepatic venous system. The surgeon alleviated CO_2_ embolism by reducing pneumoperitoneum pressure, clamping the short hepatic vein, and suturing the sieve-plate fenestrae of the middle hepatic vein. The comparison of intraoperative conditions between the two groups is presented in **Table 3**.

**Table 3:** The comparison of the intraoperative conditions of patients between groups with different inflow occlusion techniques.

LLR				
	Regional occlusion(*n* = 74)	Pringle’s maneuver occlusion(*n* = 106)	*t*/*χ* ^2^	*p*-value
Tumor location			6.341	0.096
Left lateral lobe	31	31		
Left inner lobe	11	10		
Right anterior lobe	14	34		
Right posterior lobe	18	31		
Operative time (min)	239.23 ± 96.26	204.93 ± 89.62	2.450	0.015
Blood loss (ml)	209.05 ± 181.28	292.92 ± 309.69	2.284	0.024
Blood transfusions (U)	0.33 ± 1.15	0.34 ± 1.14	0.077	0.939
Gas embolism			0.217	0.641
Negative	74	104		
Positive	0	2		

### The comparison of postoperative recovery indicators between the two groups

Liver function was re-examined on the third postoperative day to compare the intraoperative liver function damage and postoperative recovery of liver function between the two groups. We observed that ALT and AST levels were lower in the regional hepatic inflow occlusion group than in the Pringle’s maneuver occlusion group, while no statistically significant difference was observed in TBIL, albumin, and hospital days, as presented in **Table 4**.

**Table 4:** The comparison of liver function at 3 days after surgery and postoperative hospitalization days between the two groups of patients.

LLR				
	Regional occlusion(*n* = 74)	Pringle’s maneuver occlusion(*n* = 106)	*t*/*χ* ^2^	*p*-value
ALT (U/L)	105.70 ± 87.63	150.61 ± 171.95	2.066	0.040
AST (U/L)	56.05 ± 64.15	82.55 ± 91.05	2.290	0.023
Albumin (g/L)	35.96 ± 4.06	35.41 ± 4.33	0.862	0.390
Total bilirubin (μmol/L)	20.36 ± 9.57	20.17 ± 14.92	0.098	0.922
Postoperative hospitalization days	9.55 ± 3.55	9.55 ± 3.55	0.203	0.839

### The comparison of postoperative complications between the two groups

The major surgical complications of laparoscopic liver surgery include postoperative abdominal hemorrhage, bile leakage, fever, incisional infection, and ascites. Four patients in each group had bright red bloody fluid draining from the abdominal drainage tube after surgery, but all improved after conservative treatment and did not undergo reoperation. Two cases of ascites, one case of incisional infection, one case of fever, and two cases of bile leakage were observed in the regional occlusion group. One of the two patients with bile leakage had hepatocellular carcinoma, and no bile leakage was identified during intraoperative examination with the use of gauze swabs, but the bile leakage occurred after surgery. The other patient had hepatolithiasis, and the bile leakage also occurred after surgery. Five cases of ascites, one case of incisional infection, and five cases of fever occurred in the Pringle’s maneuver occlusion group, and no postoperative bile leakage occurred. There were no statistically significant differences in postoperative complications between the two groups (*χ* ^2^ = 0.111, *p* = 0.739), and all patients were discharged smoothly, as presented in **Table 5**.

**Table 5:** The analysis of postoperative complications in the two groups.

	Regional occlusion	Pringle’s maneuver occlusion	*χ* ^2^	*p*-value
Total postoperative Complication	16	25	0.228	0.954
Clavien I–II	13	21		
Clavien III–IV	3	4		
Abdominal hemorrhage	4	4		
Bile leakage	2	0		
Fever	1	5		
Incisional infection	1	1		
Ascites	2	5		
Abdominal hemorrhage + Incisional infection + Ascites	0	1		
Bile leakage + Abdominal hemorrhage	1	0		

## Discussion

Hepatectomy is still the first choice of treatment for benign and malignant liver tumors and hepatolithiasis. In recent years, with the further understanding of liver anatomy and the continuous development of laparoscopic techniques, laparoscopic hepatectomy has received increasing attention, and a history of upper abdominal surgery is no longer considered a contraindication to laparoscopic hepatectomy (7). Early laparoscopic hepatectomies were mostly localized resections of the margins or surface of the liver, and few anatomical resections were performed, and dissection of the portal vein and hepatic vein trunk was generally not required intraoperatively. Currently, with the development of technology, liver resection is now in the era of precise hepatectomy. Laparoscopy is also gradually being used in extensive hepatectomy or difficult hepatectomy (8). On one hand, the portal vein and hepatic vein are often exposed during liver surgery, and the operations of hemostasis and suture are more difficult in laparoscopic surgery than in laparotomy due to the limited operating space. On the other hand, CO_2_ pneumoperitoneum needs to be maintained for a long time, leading to gas embolism. Therefore, the two major risks of laparoscopic hepatectomy are bleeding and CO_2_ gas embolism.

Studies have shown that the incidence of conversion from laparoscopy to laparotomy due to uncontrollable intraoperative bleeding is 6%−11% (9). To reduce and control bleeding during hepatectomy, a variety of hepatic inflow occlusion techniques have been clinically used. Pringle’s maneuver is the most commonly used occlusion technique at present, with a favorable effect and a hassle-free procedure, which can be used in almost all types of hepatectomy. There are many ways of occlusion with this technique. In most patients with no adhesion or dissociable adhesions around the hepatic hilum, the hepatoduodenal ligament can be dissected, and the surgeon can use the occlusion tape to encircle the hepatoduodenal ligament for occlusion. For some patients with hepatic hilar adhesions that cannot be dissected to create a space behind the hepatoduodenal ligament, the occlusion tape cannot be passed behind the hepatoduodenal ligament. In those cases, the same favorable occlusion effect can be achieved using the Satinsky vascular clamp (10).

Despite the favorable hemostasis effect of Pringle’s maneuver, its disadvantages are noticeable as well: (1) The occlusion and subsequent restoration of hepatic blood flow easily led to hepatic ischemia–reperfusion injury; the reperfusion can activate Kupffer cells through pathways of damage-associated molecular patterns, which induce oxidative stress and inflammatory response, ultimately leading to hepatocyte injury and apoptosis (11). Pringle’s maneuver can also greatly increase systemic vascular resistance, increase pulmonary artery pressure and mean arterial pressure, reduce cardiac ejection fraction, and cause portal vein thrombosis after long-term occlusion (12). (2) Previous studies on Pringle’s maneuver have revealed that in patients without liver disease and a hepatic inflow occlusion duration of less than or equal to 20 min or patients with liver disease and a hepatic inflow occlusion duration of less than or equal to 10 min followed by 5 min of reperfusion, the liver function can recover to its preoperative level within 1−3 days. Therefore, after every 20 or 10 min of inflow occlusion, 5 min of release is required, which interrupts the continuity of the operation.

In particular, the duration of occlusion would often be prolonged when the control of intraoperative bleeding is time-consuming. The occlusion interval for restoring the hepatic inflow is usually 5 min, during which the wound bleeds more. Therefore, the total amount of bleeding will increase significantly as the operation time is prolonged and the times of interval increase (13). (3) Some studies have revealed that the occlusion of hepatic inflow cannot only lead to the impairment of liver function, but can also result in functional damage to the target organs. This is because the blood in the gastrointestinal tract cannot return to the systemic circulation through the portal vein, causing damage to the intestinal mucosal barrier and the subsequent translocation of bacterial endotoxin to extra-intestinal organs, which leads to the release of inflammatory mediators from immune cells to the target organs (14) (15) (16).

Regional inflow occlusion has been gradually applied to avoid the disadvantages of total hepatic inflow occlusion. More surgeons are now using regional hepatic inflow occlusion for laparoscopic liver surgery. The liver lobe vessels to be resected are dissected and occluded, followed by the hepatectomy. This technique can not only avoid the ischemia–reperfusion injury of the remnant liver, but also assure an unrestricted operation period and the continuity of the surgery. In this study, the postoperative liver function was better in the regional hepatic inflow occlusion group than in the Pringle’s maneuver occlusion group, but the surgical difficulty and complexity of the former are much higher than that of the latter, and experienced surgeons are needed to reduce the occurrence of complications.

There are some controversies about regional hepatic inflow occlusion. The regional occlusion approaches mainly include the intraglissonian and extraglissonian approaches. The intraglissonian approach was adopted in this study, in which the surgeon needs to open Glisson’s sheath, dissect the portal vein branches and hepatic artery branches, and block them. This is a demanding surgical operation, during which hemorrhage often occurs when the surgeon forcibly dissects the adhesions in Glisson’s sheath. However, with the development of the technique, more surgeons have been able to master this technique. Previously, surgical operation by the extraglissonian approach often led to the impairment of the hepatic parenchyma, which was prone to intraoperative bleeding and affected the laparoscopic view and operation. In recent years, with histological confirmation of the existence of the Laennec membrane, surgeons have been performing the extraglissonian occlusion through the Laennec membrane, to avoid the complex dissection of Glisson’s sheath and reduce the damage to the liver parenchyma (17). Some surgeons use simultaneous fluorescence-guided laparoscopy to perform hepatectomy, allowing more precise hepatectomies.

The main complications of laparoscopic hepatectomy are bleeding and CO_2_ embolization. In the Pringle’s maneuver occlusion group, the bleeding on the liver resection surface increases significantly due to repeated release of occlusion. In this study, the intraoperative bleeding was less in the regional occlusion group than in the Pringle’s maneuver occlusion group (209.05 ± 181.28 vs. 292.92 ± 309.69 ml, *t* = 2.284, *p* = 0.024). Compared with the Pringle’s maneuver occlusion group, the regional occlusion group only occluded the inflow of the operation area without affecting the blood supply of the remnant liver. There was no increase in bleeding of the hepatic cutting surface during the occlusion interval. Intraoperative operations may lead to the compression of the inferior vena cava, causing an increased blood flow rate and decreased pressure on the side wall of the blood vessel. This easily creates the Venturi effect that drives the aspiration of CO_2_ from the abdominal cavity into the circulation (18). The rapid accumulation of CO_2_ in the circulation can cause hypoxemia and acidosis. In serious cases, gas accumulation in the cardiac cavity results in pulmonary artery thrombosis, which is very risky. There was no significant difference in the incidence of CO_2_ embolism between the two groups in this study, because the hepatic venous system was not occluded in any occlusion techniques. During the operation, the dissection of the liver segment required the exposure of the hepatic vein. The wall of the hepatic vein is thin with many identified sieve-plate fenestrae. The vascular sieve-plate fenestrae can be easily exposed during the operation, resulting in bleeding and CO_2_ embolism. In case of CO_2_ embolism during operation, if the sieve-plate fenestra is small, it can be closed by applying absorbable hemostatic gauze or bipolar electrocoagulation. If the sieve-plate fenestra is large, it is necessary to reduce the pneumoperitoneum pressure and suture it, to control the bleeding or CO_2_ embolism effectively. In addition, the operation time was longer in the regional occlusion group than in the Pringle’s maneuver occlusion group (239.23 ± 96.26 vs. 204.93 ± 89.62 min, *t* = 2.450, *p* = 0.015), and the operator must carefully dissect each portal vein branch, hepatic artery branch, and bile duct branch in the first hepatic portal area before occluding the hepatic inflow area; therefore, the required operation time is prolonged.

Postoperative liver function is also an important indicator to evaluate the effect of the surgery. Some studies have revealed that regional hepatic inflow occlusion has a better protective effect on liver function in laparoscopic hepatectomy than Pringle’s maneuver. In this study, the results of ALT and AST of patients are better in the regional occlusion group than in the Pringle’s maneuver occlusion group on the third day after the operation, which is consistent with the results of other studies. The TBIL of patients in the regional occlusion group was higher than that of patients in the Pringle’s maneuver occlusion group before the operation, but there was no significant difference between the two groups after an operation, which showed that the TBIL recovery of patients in the regional occlusion group was better than that in the Pringle’s maneuver occlusion group.

There were many postoperative complications in liver surgery, and there were no significant differences in the occurrence of various complications between the two groups (10 vs. 15, *χ* ^2^ = 0.111, *p* = 0.739). Among them, postoperative bile leakage (POBL) is one of the main complications, with an incidence of about 3.6%−11% (19), and POBL will have an impact on the postoperative course (20). A study included 13,379 patients who underwent laparoscopic hepatectomy or open hepatectomy, in which the incidence of POBL in laparoscopic hepatectomy was lower than that in open hepatectomy (21). There are many reasons for the occurrence of bile leakage. First, some intraoperative bile leakage locations are difficult to operate under laparoscopy; therefore, it is impossible to close the bile duct effectively. Second, some are delayed bile leakage that cannot be identified despite a careful inspection of the operator on the hepatic cutting surface during the operation. There were two cases of POBL in the regional occlusion group and one case of POBL in the Pringle’s maneuver occlusion group, and all of them had hepatolithiasis, which may be related to local inflammation of the bile duct (22), bile duct dilatation, or ineffective coagulation of the bile duct by an ultrasonic knife. Bile leakage can cause infection; the bacterial culture of abdominal drainage fluid from the patient was positive, indicating abdominal infection. The patient was cured after puncture drainage and was discharged smoothly. There were more than 100 ml of bright red drainage fluid in the drainage tube of eight patients in the two groups after an operation. After applying hemostatic medication and prothrombin complex, all the patients were discharged smoothly. Moreover, complications were also related to the operator’s experience in laparoscopic hepatectomy.

In conclusion, this study suggests that Pringle’s maneuver is suitable for patients with a small hepatic cutting surface after resection and anticipated short operation time. Regional hepatic inflow occlusion has the advantages of better continuity of the operation, less intraoperative hemorrhage, and less damage to liver function. The long duration and high precision of the regional hepatic inflow occlusion technique demands a more experienced physician with a higher level of operation; therefore, it can be performed by experienced laparoscopic liver surgeons.

## Data availability statement

The raw data supporting the conclusions of this article will be made available by the authors, without undue reservation.

## Author contributions

LS and XL conceived and designed the study as well as drafted the manuscript. BL and YM collected the data. YY and DS conducted the statistical tests. QZ made language changes. All authors read and approved the final manuscript.

## Funding

This work was supported by the Natural Science Foundation of Jiangsu Province (BK20190138), Changzhou Society Development Funding (CE20205038).

## Conflict of interest

The authors declare that the research was conducted in the absence of any commercial or financial relationships that could be construed as a potential conflict of interest.

## Publisher’s note

All claims expressed in this article are solely those of the authors and do not necessarily represent those of their affiliated organizations, or those of the publisher, the editors and the reviewers. Any product that may be evaluated in this article, or claim that may be made by its manufacturer, is not guaranteed or endorsed by the publisher.
